# Preparation and Functional Identification of a Novel Conotoxin QcMNCL-XIII0.1 from *Conus quercinus*

**DOI:** 10.3390/toxins14020099

**Published:** 2022-01-26

**Authors:** Han Zhang, Anwen Liang, Xinghua Pan

**Affiliations:** 1 Department of Biochemistry and Molecular Biology, School of Basic Medical Sciences, Southern Medical University, Guangzhou 510515, China; 2 Guangdong Provincial Key Laboratory of Single Cell Technology and Application, Southern Medical University, Guangzhou 510515, China; 3 Guangdong-Hong Kong-Macao Greater Bay Area Center for Brain Science and Brain-Inspired Intelligence, Southern Medical University, Guangzhou 510515, China; 4 School of Life Sciences, Sun Yat-Sen University, Guangzhou 510006, China; lianganw@mail2.sysu.edu.cn

**Keywords:** *Conus*, conotoxin, pethidine, α1G, CACNA1G, T-type calcium channel

## Abstract

Conotoxins are tools used by marine *Conus* snails to hunt and are a significant repository for marine drug research. Conotoxins highly selectively coordinate different subtypes of various ion channels, and a few have been used in pain management. Although more than 8000 conotoxin genes have been found, the biological activity and function of most have not yet been examined. In this report, we selected the toxin gene QcMNCL-XIII0.1 from our previous investigation and studied it in vitro. First, we successfully prepared active recombinant QcMNCL-XIII0.1 using a TrxA (Thioredoxin A)-assisted folding expression vector based on genetic engineering technology. Animal experiments showed that the recombinant QcMNCL-XIII0.1 exhibited nerve conduction inhibition similar to that of pethidine hydrochloride. With flow cytometry combined fluorescent probe Fluo-4 AM, we found that 10 ng/μL recombinant QcMNCL-XIII0.1 inhibited the fluorescence intensity by 31.07% in the 293T cell model transfected with Cav3.1, implying an interaction between α1G T-type calcium channel protein and recombinant QcMNCL-XIII0.1. This toxin could be an important drug in biomedical research and medicine for pain control.

## 1. Introduction

Although hundreds of toxins have been found, very few have been successfully prepared and tested for their nerve-blocking activity [1,2,3]. The anesthetic effect and the pharmacological activity mainly depend on the specific conotoxin employed [1,2,3].

The action targets of conotoxin predominantly fall into three categories: (1) voltage-gated ion channels, including Na^+^, K^+^, and Ca^2+^ channels, typified by conotoxin acting on the K^+^ channels κ-PVIIA [4]; (2) ligand-gated ion channels, including acetylcholine receptor and serotonin receptor (5-HT3R), exemplified by α4/7-CTx LvIA, which is a new, potent, and selective α3β2 nAChR antagonist [5]; and (3) G-protein-coupled receptors, such as conulotoxin Contulakin-G, which can activate G-protein-coupled angiotensin receptors [6,7]. A single conotoxin molecule often has strong target specificity, can specifically coordinate cell membrane ion channels or receptors, and can even specifically distinguish between different subtypes of receptors [8,9,10,11,12,13]. Conotoxin highly selectively coordinates different subtypes of various ion channels [14] and ziconotide (sold as Prialt^®^) has been used in the treatment of pain [15]. There are numerous conotoxins that have been shown preclinically to have an effect (in vitro or in vivo in animal models) [6,16,17,18,19].

T-type Ca^2+^ channels mainly include a single pore-forming α1 subunit and belong to the family of voltage-gated calcium channels (VGCCs), which control calcium ion flux across the cell membrane and are known sites of channel regulation by secondary messengers, drugs, and toxins. Three genes, *CACNA1G*, *CACNA1H*, and *CACNA1I*, encode for the three T-type channel isoforms Cav3.1 (α1G), Cav3.2 (α1H), and Cav3.3 (α1I), respectively, in humans [20,21,22]. The T-type Ca^2+^ channel belongs to the calcium channel family, which is activated by low voltage, and when current is short and weak, it can be deactivated rapidly [23,24]. Due to its small conductance, the T-type Ca^2+^ channel is thought to be related to pacemaker activity, low threshold calcium peaks, neuronal oscillation and resonance, and rebound pulse discharge [25]. T-type Ca^2+^ channels exist not only in the heart and smooth muscles but also in many neurons in the central nervous system. The influx of calcium into cells can cause cells to produce a variety of physiological responses [26,27,28,29]. In cardiomyocytes and smooth muscle cells, an activated voltage calcium channel and the increase in calcium solute concentration directly lead to cell contraction, which in turn leads to tissue contraction [30,31]. These calcium channel families have been identified to be highly expressed in the brain, the peripheral nervous system, heart, smooth muscles, bones, and the endocrine system [32]. Modulating the activity of alpha1G subtype channels may provide a novel approach for the treatment of allodynia and hyperalgesia [33,34]. At present, there are four approved T-type channel modulators, namely ethosuximide (zarontin^®^), valproic acid (Depakene^®^) and zonizamide (Zonegrans^®^), blockers, and L-type channel blockers. These inhibitors all works on Ca^2+^ channel Cav3.2, while there are few products that work on Cav3.1 regulators [35,36].

Here, based on our previous investigation of *Conus quercinus*, using a prokaryotic expression vector with TrxA (Thioredoxin A), we successfully constructed a new toxin gene QcMNCL-XIII0.1 (Supplementary Table S3 [37], named Que-2-c57915_g1_5_6), which induced the high expression, purification, detection, and identification of the toxin. By the improved method of bullfrog sciatic nerve bioelectric activity, we defined its biological nerve-blocking activity, and by flow cytometry combined with fluorescent probe Fluo-4 AM, we preliminarily studied the function of QcMNCL-XIII0.1 in the 293T cell model.

## 2. Results

### 2.1. Expression, Purification, and Identification of QcMNCL-XIII0.1

**Figure 1:**
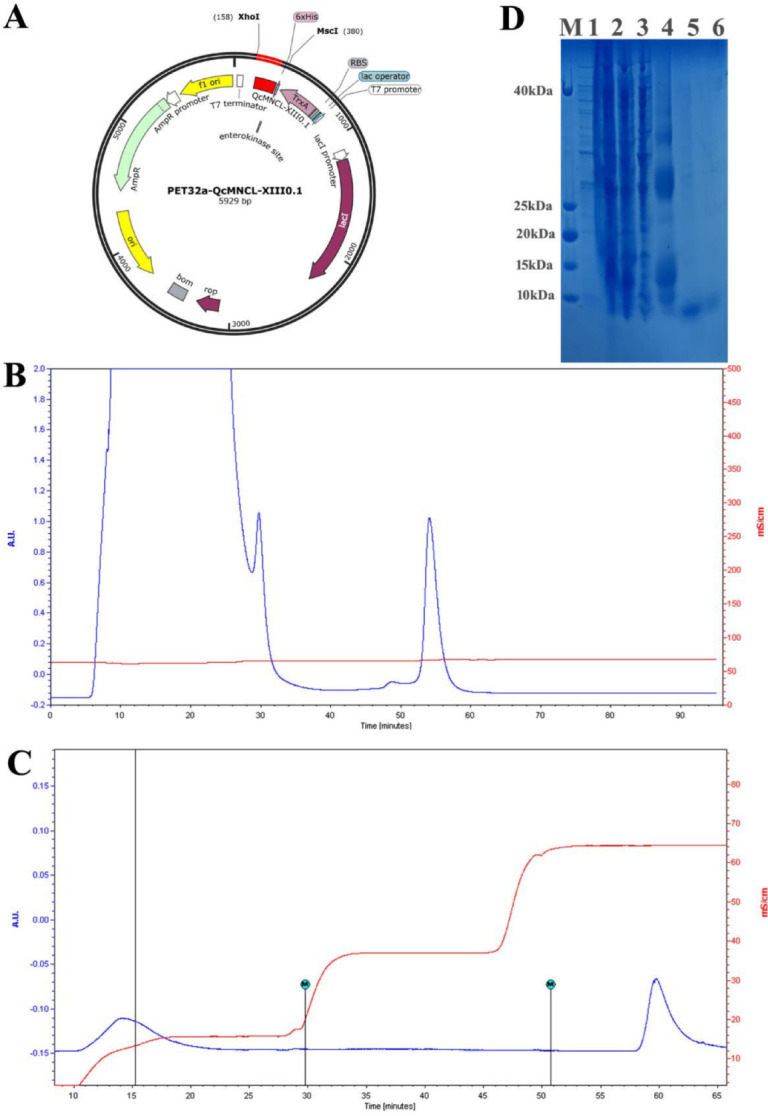
Purification of recombinant QcMNCL−XIII0.1: (**A**) the expression plasmid PET32a-QcMNCL−XIII0.1; (**B**) Ni^2+^-chelating sepharose fast-flow chromatography of TrxA-QcMNCL-XIII0.1; (**C**) Ni^2+^-chelating sepharose fast-flow chromatography of QcMNCL−XIII0.1; (**D**) SDS–PAGE analysis recombinant QcMNCL−XIII0.1 expression. M: standard protein marker; 1: uninduced supernatant from BL21 (DE3) cells; 2: T and total protein from BL21 (DE3) cells induced with IPTG; 3: supernatant from BL21 (DE3) cells induced with IPTG after ultrasonification; 4: washed protein from Ni^2+^-chelating sepharose chromatography in elution buffer; 5 and 6: sample of the fusion protein QcMNCL−XIII0.1.

The sequencing results of the fusion expression plasmid (Figure 1A) were consistent with the expected results (Supplementary Document S1), that is, the reading frame was correct and the resulting protein was full length. The final induction conditions for the expression of the target protein were as follows: when the OD_600_ was 0.5 at 37 °C, IPTG at a final concentration of 0.1 mM was added, the cells were cultured at 18 °C, and protein was induced for 8 h. The total proteins were purified and analyzed before enzymatic digestion. According to the order of peak time, the two peaks were TrxA-6×His-QcMNCL-XIII0.1 and nontarget peptides (Figure 1B).

**Figure 2:**
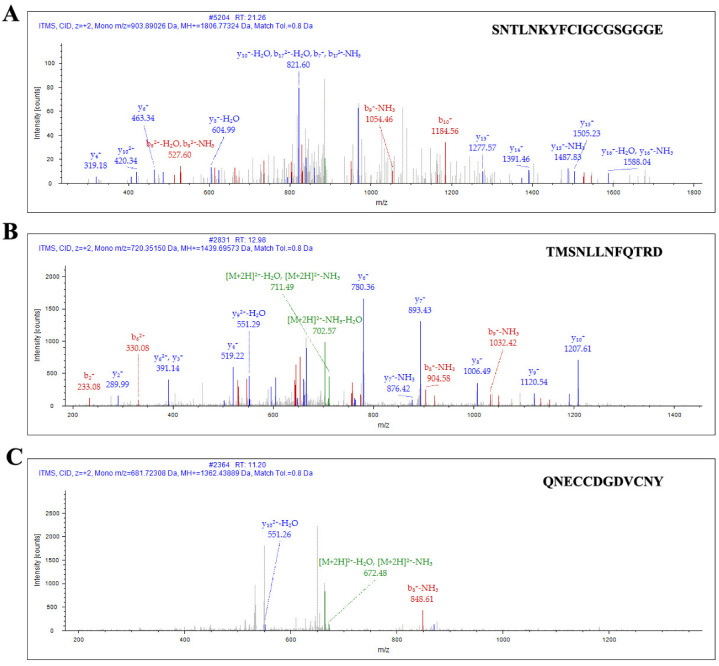
Ion spectrum of QcMNCL−XIII0.1 by LC–MS/MS. (**A**) Identified sequence of QcMNCL−XIII0.1: SNTLNKYFCIGCGSGGGE; (**B**) identified sequence of QcMNCL−XIII0.1: TMSNLLNFQTRD; (**C**) identified sequence of QcMNCL−XIII0.1: QNECCDGDVCNY.

After enzyme digestion, affinity purification was again carried out. According to the order of peak time, the two peaks from left to right were QcMNCL-XIII0.1 and a non-target peptide with a 6 ×His tag (Figure 1C). SDS–PAGE (sodium dodecyl sulfate polyacrylamide gel electrophoresis) before and after peptide purification is shown in Figure 1D. The amino acid sequence of QcMNCL-XIII0.1 is TMSNLLNFQTRDCPSSCPAVCPNQNECCDGDVCNYSNTLNKYFCIGCGSGGGE. Cysteine amino acids may be connected in many ways within the molecule. The theoretical mass of QcMNCL-XIII0.1 was 5.63 kDa, meaning that the molecular weight closely matched the theoretical weight. The QcMNCL-XIII0.1 peptides SNTLNKYFCIGCGSGGGE (Figure 2A), TMSNLLNFQTRD (Figure 2B), and QNECCDGDVCNY (Figure 2C) were also successfully identified by LC–MS/MS mass spectrometry.

### 2.2. The Biological Activity of QcMNCL-XIII0.1

**Figure 3:**
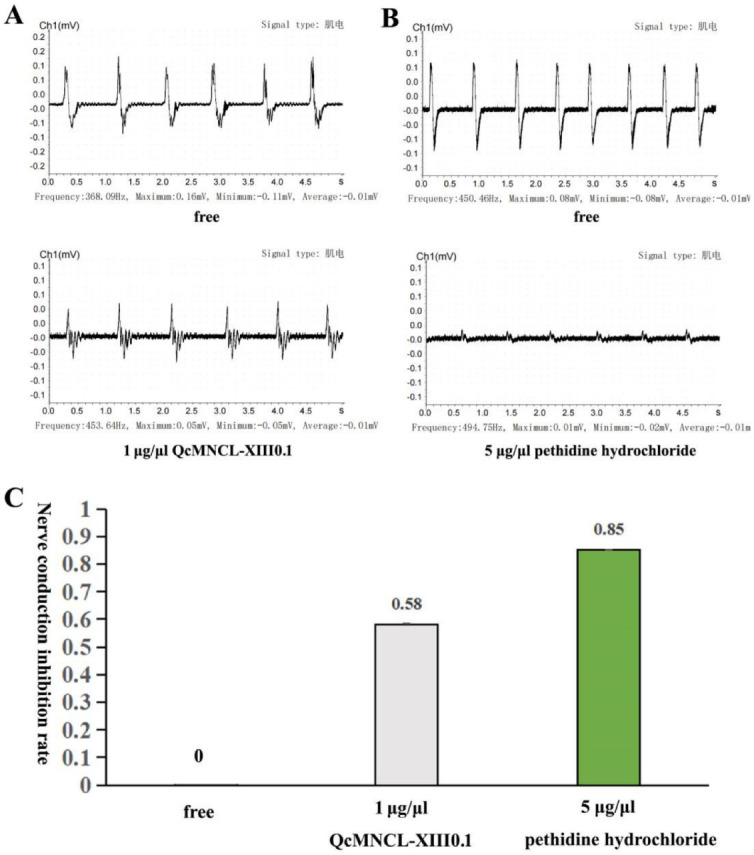
Inhibitory action of QcMNCL−XIII0.1 and on neuromuscular transmission. (**A**) A total of 1 μg/μL of QcMNCL-XIII0.1; (**B**) a total of 5 μg/μL of pethidine hydrochloride (reanalysis from our published data [37]); (**C**), Nerve conduction inhibition rate of QcMNCL−XIII0.1 and pethidine hydrochloride.

We designed a model to test the biological activity of recombinant QcMNCL-XIII0.1, as shown in Figure 6 in another study [37] and as described in the Materials and Methods. We mainly used the BL-420S biological function experimental system (Chengdu Techman Software Co. Ltd.) to convert the strength of the frog nerve reflex signal into an electromyography (EMG) signal. According to our experimental model, six electrical stimulations were performed and six signals were collected each time. Each electrical stimulation (each peak) was considered a repeated experimental test. We recorded six electrical stimulation signals before drug administration and six when 1 μg/μL recombinant QcMNCL-XIII0.1 was applied. Based on the statistical methods in the Materials and Methods section, we found that 1 μg/μL recombinant QcMNCL-XIII0.1 inhibited the neural signal transduction by 58% (Figure 3A). Pethidine was used as a positive control. In our previous investigation, we again found that 5 μg/μL pethidine hydrochloride inhibited the neural signal transduction to 85% (Figure 3B and Supplementary Figure S1). The recombinant QcMNCL-XIII0.1 prepared showed nerve conduction inhibition similar to that of anesthetics.

### 2.3. Construction of Eukaryotic Expression Vector for the CACNA1G Gene and Identification of the Expression of α1G Membrane Protein

Conotoxin QcMNCL-XIII0.1 showed a high inhibition rate of neuromuscular conduction, and its application prospects are great; therefore, we further explored its possible targets. We chose to investigate whether conotoxin QcMNCL-XIII0.1 could affect the activity of Cav3.1 channels in cell membranes by measuring the changes in intracellular calcium concentration before and after the administration of the toxin.

As described in the Materials and Methods section, the successful construction of the pcDNA3.1-CACNA1G-ceGFP vector (Supplementary Figure S1A) was confirmed by sequencing. The sequence peak diagram is shown in Supplementary Document S2.

Since the three terminals of the *CACNA1G* vector had a *ceGFP* fluorescent protein tag, the expression of membrane protein could be observed by ce*GFP*. Our experimental observations show that the membrane protein α1G was successfully expressed in 293T cells, and fluorescence was observed after 24 h (Supplementary Figure S1B) and 36 h (Supplementary Figure S1D). No fluorescence was detected in cells not transfected with the plasmid (Supplementary Figure S1C). The expression of ce*GFP* was also successfully detected by flow cytometry, and the transfection efficiency was approximately 18% (Supplementary Figure S1E).

**Figure 4:**
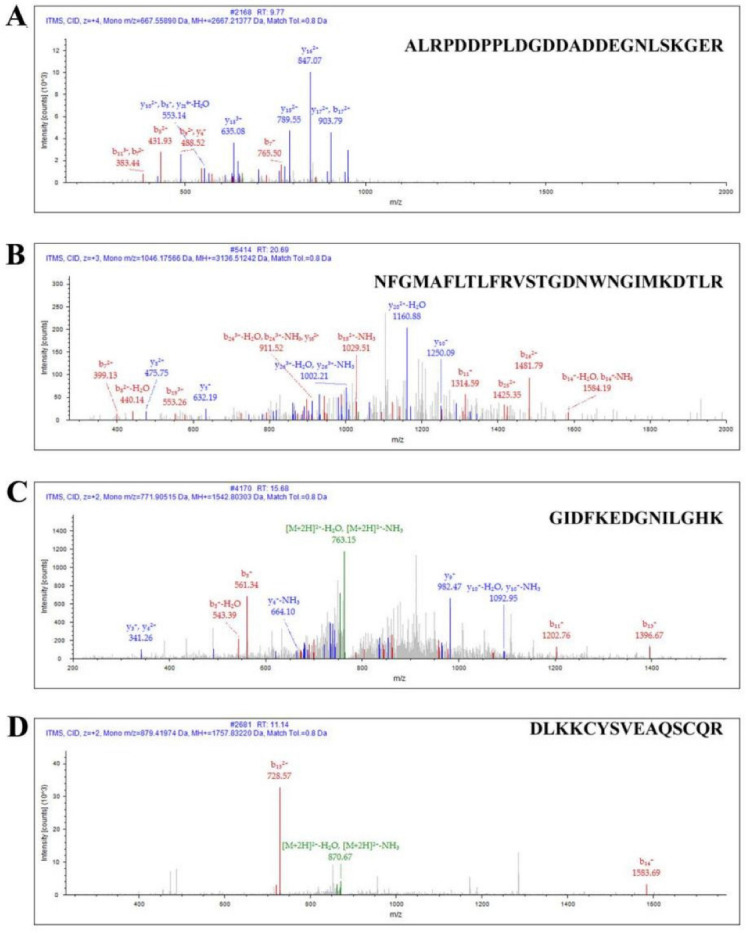
Identification of CACNA1G-ceGFP protein by LC–MS/MS. (**A**) Ion spectrum of identified sequence: ALRPDDPPLDGDDADDEGNLSKGER; (**B**) ion spectrum of identified sequence: NFGMAFLTLFRVSTGDNWNGIMKDTLR; (**C**) ion spectrum of identified sequence of ceGFP: GIDFKEDGNILGHK; (**D**) ion spectrum of identified sequence of ceGFP: DLKKCYSVEAQSCQR.

Then, these cell membrane proteins were extracted and assessed by mass spectrometry. The α1G membrane protein sequences expressed by *CACNA1G* were successfully identified by LC–MS/MS to be ALRPDDPPLDGDDADDEGNLSKGER (Figure 4A) and NFGMAFLTLFRVSTGDNWNGIMKDTLR (Figure 4B). In addition, one *GFP* fluorescent protein tag sequence was successfully identified: GIDFKEDGNILGHK (Figure 4C) and DLKKCYSVEAQSCQR (Figure 4D). Therefore, we successfully expressed the exogenous α1G membrane protein in 293T cells.

### 2.4. Effect of QcMNCL-XIII0.1 on α1G Membrane Proteins

**Figure 5:**
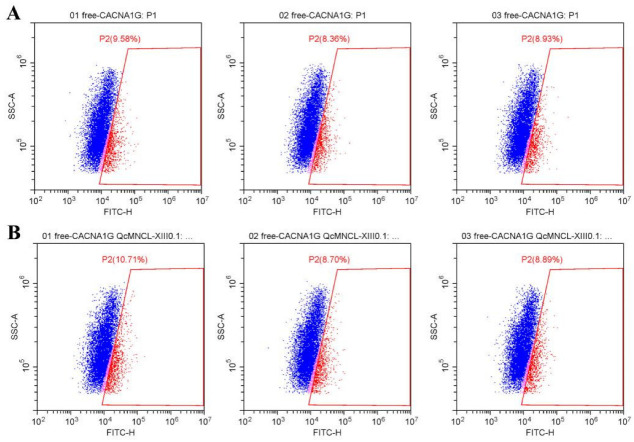
Effect of the fluorescence intensity on free 293T cells without QcMNCL-XIII0.1 or Cav3.1 plasmid: (**A**) free; (**B**) recombinant 10 ng/μL QcMNCL-XIII0.1.

Fluo-4 AM was used as a fluorescent probe to detect intracellular calcium concentration changes, as it can penetrate the cell membrane and be taken up by cells. These changes in intracellular calcium ion concentration were then measured by flow cytometry. We found that conotoxin QcMNCL-XIII0.1 demonstrated little effect on intracellular calcium ion concentration in 293T cells without plasmid transfection before (Figure 5A) and after administration (Figure 5B), and the fluorescence intensity was basically unchanged or had no significant difference.

**Figure 6:**
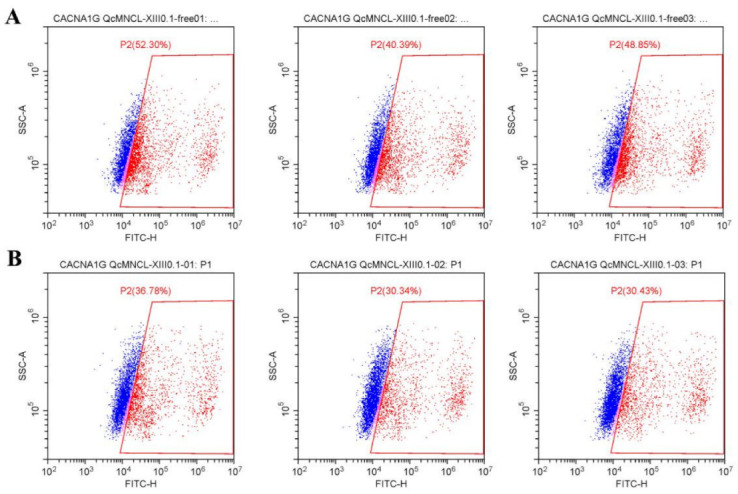
Effect of the fluorescence intensity on free 293T cells after transfection with Cav3.1-ceGFP plasmid: (**A**) free; (**B**) recombinant 10 ng/μL QcMNCL-XIII0.1.

However, after the transfection of *CACNA1G* with a GFP fluorescent label and the addition of the Fluo-4 fluorescent probe, the fluorescence intensity doubled, indicating that the probe was successfully loaded (Figure 6A). By examining populations of cells before and after the addition of 10 ng/μL conotoxin QcMNCL-XIII0.1, we found that the percentage inhibition was (47.18–32.52%)/47.18%) * 100%, which was a 31.07% inhibition compared with the buffer control (Table 1 and Figure 6B). The difference between the D and E groups was statistically significant, and the *p*-value of the t test was 0.0238. This indicated that the conotoxin QcMNCL-XIII0.1 caused the outflow of calcium ions from cells, resulting in a decrease in intracellular calcium ion concentration. We thus speculated that QcMNCL-XIII0.1 directly or indirectly causes intracellular calcium outflow through the T-type calcium channel α1G.

## 3. Discussion

Although hundreds of conotoxins have been found [5,38,39,40,41], very few of them have been successfully prepared and tested for nerve-blocking activity [42,43,44]. The chemical synthesis of peptides with more than three pairs of disulfide bonds is difficult [45]. After a series of trials, we obtained high purity of the conopeptide QcMNCL-XIII0.1 by constructing an expression system with the tag TrxA. In the design of an expression vector, we skillfully used the enterokinase with this label for enzyme digestion to facilitate the downstream purification and recovery of the target polypeptide. On average, 5.25 mg of recombinant QcMNCL-XIII0.1 conotoxin was obtained from 1 L culture. According to the prediction results on the ConoSever, the conotoxin QcMNCL-XIII0.1 may contain four pairs of disulfide bonds, while the other two conotoxins (κ-PVIIA [46] and GeXIV [47]) have only three pairs of disulfide bonds. This may be the key element responsible for the special features of QcMNCL-XIII0.1, such as stability. Animal tests showed that recombinant QcMNCL-XIII0.1 had a strong inhibitory effect on neural signal transduction and nerve conduction inhibition similar to that of anesthetics. The abuse of pethidine-containing drugs is frequently encountered in patients [48]. The conotoxin QcMNCL-XIII0.1, which can block nerve signals at low doses, may become an alternative drug in the future.

In addition, in analyzing sequence homology on the ConoSever, we found that QcMNCL-XIII0.1 overlaps with the peptide conoCAP-a (a CCAP-related peptide) [49] of the crustacean cardioactive peptide (CCAP) superfamily by 90%, which is a surprising discovery and which provides guidance for further experimentation. Later studies found CCAP to be a multifunctional neuropeptide involved in several physiological processes, such as the release of adipokinetic hormone from the corpora cardiaca and modulating the cardiac ganglion [50,51]. The application of conoCAP-a on rat cardiac myocytes gradually decreased systolic Ca^2+^ transient amplitude, but conoCAP-a had no effect on L-type Ca^2+^ channel (LTCC) current and other cell membrane channels and receptors involved in the cardiovascular physiology (β1-AR, β2-AR, HCN1, HCN2, Kv1.4, Nav1.5, hERG, V1a, V1b, and V2) [49]. In the pre-experiment, we also tested the cell models transfected with alpha4 nicotinic receptor (*CHRNA4*), alpha6 nicotinic receptor (*CHRNA6*), alpha7 nicotinic receptor (*CHRNA7*), and alpha9 nicotinic receptor (*CHRNA9*) and found that there was no change evident in the calcium concentration of cells. Therefore, we studied the effect of QcMNCL-XIII0.1 on cells transfected with T-type calcium channel protein. T-type Ca^2+^ channels play an essential role in the functioning of the nervous system [52]. In addition, T-type calcium channels are a significant etiological factor of pain for a variety of cancers and mainly control and regulate the proliferation, survival, and cell cycle of cancer cells [11,53,54,55,56,57,58,59,60,61]. However, there are few reports of selective regulators of T-type calcium channel protein Cav3.1 (α1G). In the 293T cell model, our experimental results show that the calcium concentration was inhibited by 31.07% after the QcMNCL-XIII0.1 was added. Our results suggest that the QcMNCL-XIII0.1 maybe interact with Cav3.1 (α1G) to inhibit intracellular calcium concentration. Single amino acid variations strongly affect its activity and function [49]. This study focuses on the natural origin of conotoxins, and we will continue to study variants in the future.

In our experiment, Fluo-4 AM was used to compare calcium levels in same populations of cells. Fluo-4 is well suited for photometric and imaging applications that make use of confocal laser scanning microscopy and flow cytometry [62,63,64]. For the reliability of our experiment, we designated as blank controls groups A and B, and blank group C (Table 1). The continuity of the experimental results preliminarily supports our results. In the future, we may use red or blue fluorescent protein labels for further verification or even a patch clamp experiment.

## 4. Conclusions

We prepared high-purity samples of the conopeptide QcMNCL-XIII0.1 by genetic engineering. Recombinant QcMNCL-XIII0.1 exhibits the ability to block nerve signal transduction in an ex vivo model, similar to that of the anesthetic pethidine hydrochloride. Based on cell flow cytometry, we preliminarily confirmed that the fluorescence was inhibited by 31.07% in the 293T cell model transfected with Cav3.1, implying the interaction between α1G T-type calcium channel protein and recombinant QcMNCL-XIII0.1. The discovery of QcMNCL-XIII0.1 is conducive to the follow-up study of T-type calcium channel protein Cav3.1 (α1G) and promotes its research in the study of pain and in other fields.

## 5. Materials and Methods

### 5.1. Construction of Recombinant Expression Vector

According to the nucleotide sequence of the mature peptide encoded by the conotoxin gene, combined with the multiple cloning site of PET32a (+) vector and the restriction endonuclease site of toxin gene, the codon of the toxin gene was optimized for expression in *E. coli*. The toxin gene was short, and it was synthesized by direct synthesis. The vector design map is shown in Figure 1. The key steps were as follows: (1) A 50 μL enzyme digestion system was used, composed of 1 μL of *KpnI*, 1 μL of *XhoI*, 5 μL of 10×NEB buffer 1.1, 1 μg of toxin gene fragment, and 43 μL of deionized water. Enzyme digestion at 37 °C in a constant temperature water bath was performed for 12 h. (2) The pGEM^®^-T Easy Vector System I was used for plasmid construction (Promega Corporation). The screening of engineered strains was performed using T7 primers, the first generation of clones was sequenced using an ABI3730XL gene sequencer, and positive were clones identified. After the correct construction of the extracted plasmid was confirmed by sequencing, the bacteria were preserved at −80 °C. (4) Recombinant expression in vitro was performed using strains cultured in 2YT medium containing ampicillin at 37 °C for 2–3 h until OD_600_ = 0.55–0.75, and then, expression was induced by IPTG at 18 °C for 10 h. We separately collected the uninduced supernatant of BL21 (DE3) cells, the supernatant from BL21 (DE3) cells induced by IPTG after ultrasonification, and the wash protein from Ni^2+^-chelating sepharose in elution buffer, and all were verified using 12% sodium dodecyl sulfate polyacrylamide gel electrophoresis (SDS–PAGE) analysis.

### 5.2. Purification of Recombinant Conotoxin

The purification process for recombinant conotoxin was as follows: (1) Single colonies were cultured in 15 mL of 2YT medium with shaking at 37 °C for 12 h as seed cultures. At a ratio of 1:50, they were inoculated in 1 L of 2YT medium and cultured at 37 °C until the OD_600_ value reached 0.6–0.7. At a ratio of 1:1000, 24 mg/mL of IPTG and 5% of glucose solution were added, and the cells were cultured at 18 °C for another 10 h. (2) The bacteria were collected after centrifugation at 4 °C and 8000 rpm for 10 min. The supernatant was collected after ultrasonication for 1 h at 4 °C by centrifugation at 12,000 rpm for 20 min. The fusion toxin polypeptides containing TrxA, a histidine label, and an EK digestion site were isolated by Ni affinity chromatography. (4) The fusion protein solution was passed as 4 mL through a 3K ultrafiltration column and replaced with EK digestion buffer. (5) Then, 1% EK enzyme with a histidine tag was added, and the proteins were digested at 22 °C for 18 h. After digestion, the solution was passed through a Ni-affinity chromatography column, the first peak was detected, and the target conotoxin was isolated. The size of the Ni^2+^-chelating sepharose affinity chromatography column was 1.6 cm × 4 cm. A constant flow rate of 2 mL/min was maintained throughout the operation, and a Tris-HCl buffer system was used. The ethanol-sealed Ni^2+^-chelating sepharose affinity chromatography column was loaded with deionized water, and 0.2 M NiSO_4_ was loaded to bind the Ni^2+^ to the column material. Next, we used deionized water to remove the uncombined NiSO4, and this was balanced with an ultrasonic buffer (50mM Tris, 0.5M NaCl, and 20 mM imidazole, pH 8.0). The supernatant on the column after ultrasonication was washed with ultrasonic buffer until the UV absorption value reached its baseline, and then, a gradient elution was carried out with a mixture of ultrasonic buffer and elution buffer (50 mM Tris, 500 mM NaCl, and 500 mM imidazole, pH 8.0).

### 5.3. Protein Fractionation, Preparation for LC–MS/MS Analysis, and Proteomic Data Analysis

The LC–MS/MS methods are summarized in our previous articles [37]. The total cell membrane proteins from 293T cells were solubilized in lysis buffer (8 M urea, pH 8.00) containing 5 mM DTT at 60 °C for 45 min, followed by alkylation at 25 °C for 45 min in the presence of 25 mM iodoacetamide in the dark. The obtained protein solutions were reconstituted in 50 mM ammonium bicarbonate with 0.5 M urea, pH 7.8, and digested (trypsin: protein = 1:100) for 12 h at 37 °C. The test time of the total cell membrane proteins was 50 min. The test time of purified recombinant polypeptide sample was 30 min.

### 5.4. Bullfrog Sciatic Nerve Bioelectric Activity

In this experiment, bullfrog sciatic nerve bioelectric activity was used to assess whether the expressed conotoxin had the effect of blocking neuromuscular conduction [65,66,67]. According to our previous published study [37], animal experimental models were prepared and are shown in the Graphical Abstract of this paper. The tension transducer and the stimulation electrode were connected to a BL-420S biological function experimental system (Chengdu Techman Software Co. Ltd., Chengdu, China). The concentration of conotoxin was 1 μg/μL. The dosage was 50 μL. In the calculation of the experimental results, six peaks were collected, and the peak and the trough of each extracted. The peak represents the maximum pulling force. Inhibition rate of neural circuit = (value before administration-value after administration/value before administration) * 100%. Value = peak − trough.

### 5.5. Construction of pcDNA3.1-CACNA1G-ceGFP Vector

The cDNA template was donated by the Han Jiahuai Laboratory of Xiamen University. PCR amplification was carried out according to established methods. First, the T easy vector was constructed, and then, the eukaryotic expression vector was constructed. Moreover, 5′-terminal *EcoR I* and 3′ *Not I* sites were added.

According to the full-length sequence of the spliced gene, the primers were designed using PREMIER5.0 software and the restriction sites were introduced to construct the eukaryotic expression vector pcDNA3.1-CACNA1G-ceGFP. The sequencing primers used were the T7 promoter (ATTATGCTGAGTGATATCCC) and the SP6 promoter (TAAGATATCACAGTGGATTTA). The full-length primer for the *CACNA1G* gene was CACNA1G-Up primer (ATGGACGAGGAGGAGGATGGAGC) and CACNA1G-Low (primer GGGGTCCAGGTCTGCTGGGTCAGAGGATAA). We performed PCR product recovery, ligation, transformation, and positive colony screening in turn. Plasmid extraction was performed using the Plasmid Mini prep Kit II kit from Omega Bio-Tek, and monoclonal positive plasmids were extracted according to the manufacturer’s instructions. Sequencing of the target gene was performed at Beijing Tsingke Biotechnology Co., Ltd. and Guangzhou IGE Biotechnology Ltd. The obtained sequence was opened by UGENE, saved as an FASTA format document and then assembled using the Seqman module in DNASRAT. The sequence file was modified according to the specific sequence atlas, and then, sequence analysis was carried out.

After the pcDNA3.1-CACNA1G-ceGFP monoclonal recombinant vector was obtained, an endotoxin-free plasmid was extracted using the OMEGA Endo-free Plasmid mini kit (OMEGA) and the specific operation process was carried out according to the manufacturer’s instructions.

### 5.6. Transient Transfection of Recombinant Plasmid into 293T Cells

Cell strains were stored in liquid nitrogen. After removal, they were immediately incubated in a 37 °C water bath and then transferred to DMEM medium containing 10% fetal bovine serum. Twelve-well plates were used in these experiments. Generally, after 3–4 passages, when the cells were in good condition, they were transfected. Cells were seeded at a cell density of approximately 1 × 10^5^ cells per well. After incubation overnight, the cells were completely adhered to the plates, and the density reached approximately 60–80%; therefore, transfection was carried out. During transfection, in addition to the transfection of the constructed fusion vector, it was also necessary to transfect an empty vector as a blank control. The transfection experiment was carried out according to the Lipofectamine™ 3000 Transfection Reagent (Invitrogen, Carlsbad, CA, USA) instructions.

### 5.7. Extraction of Membrane Proteins and Identification by Mass Spectrometry

Total cell membrane proteins were extracted using the Minute™Plasma Membrane Protein Isolation and Cell Fractionation Kit (Invent Biotechnologies, Eden Prairie, MN, USA). The 293T cells cultured for 36 h after plasmid transfection were used as the experimental group, while untreated 293T cells were used as the blank group to extract the total cell membrane proteins from 5 × 10^5^ 293T cells. Then, mass spectrometry samples from the total cell membrane proteins were prepared for LC–MS/MS.

### 5.8. Detection of Intracellular Calcium Concentration by Flow Cytometry

Preparation of Fluo-4 AM (CAS No.: 273221-67-3) stock liquor was achieved by adding an appropriate amount of 330 µL of DMSO to Fluo-4 AM to prepare 400 μM of storage solution that was stored away from light. Fluo-4 AM working solution was made at 1:100, to avoid repeated freeze–thaws. A negative control of just DMSO was used in these experiments. Each experiment was repeated three times. The 293T cells were transiently transfected for 36 h, the culture medium was removed, and the cells were washed with HBSS solution three times. The Fluo-4 AM working solution was added to the cells, adding enough to cover the cells, and the cells were incubated at 37 °C at 5% CO_2_ for 45 min. Next, the Fluo-4 AM working solution was removed, and the cells were re-suspended in HBSS solution. The cells were incubated for another 10 min and gently shaken several times to ensure the complete de-esterification of AM bodies in the cells. The cells were divided into two groups, each with three replicates. The experimental group was treated with 10 ng/μL QcMNCL-XIII0.1, and the control group was treated with the same amount of HBSS, the total amount of which was the same as the volume of QcMNCL-XIII0.1 added. The cells were placed in 5% CO_2_ in the dark at 37 °C for 1 min. The cells were then immediately assessed by flow cytometry. The excitation wavelength was 494 nm, and the emission wavelength was 516 nm. This spectrum basically overlaps with that of FITC, so the spectrum detection channel was set as FITC. A total of 10,000 cells were collected: 5000 cells were collected both before and after administration. The intracellular calcium concentration was expressed as the average fluorescence, and the rate of change in fluorescence was calculated. The percentage inhibition of the fluorescence change rate = (administration group − control group)/administration group × 100%.

## Figures and Tables

**Table 1:** The fluorescence intensity of 293T cells. Note: A, 293T cells + free + Fluo-4 (Figure 5A); B, 293T cells + free + Fluo-4 + 10 ng/μL QcMNCL-XIII0.1 (Figure 5B); C, 293T cells transfection with GFP plasmid in 36 h (Supplementary Figure S1E); D, 293T cells transfection with *Cav3.1-ceGFP* plasmid in 36 h + Fluo-4 (Figure 6A); E, 293T cells transfection with *Cav3.1-ceGFP* plasmid in 36 h + Fluo-4 + 10 ng/μL QcMNCL-XIII0.1 (Figure 6B). In theory, 100% fluorescence means that the cells detected by flow cytometry were labeled with fluorescent protein *ceGFP* or Fluo-4.

	A	B	C	D	E
	9.58%	10.71%	18.26%	52.30%	36.78%
	8.36%	8.70%	18.34%	40.39%	30.34%
	8.93%	8.89%	18.95%	48.85%	30.43%
Average	8.96%	9.43%	18.52%	47.18%	32.52%
Variance	0.00%	0.01%	0.00%	0.38%	0.14%

## Data Availability

Not applicable.

## Supplementary Materials

The following supporting information can be downloaded at: https://www.mdpi.com/article/10.3390/toxins14020099/s1, Supplementary Figure S1: The 293T cells transfected with pcDNA3.1-CACNA1G-ceGFP detected by GFP fluorescence microscopy. (A) The map of pcDNA3.1-CACNA1G-ceGFP; (B) fluorescence microscopic observation after 24 h; (C) positive control group; (D) fluorescence microscopic observation after 36 h; (E) flow cytometry fluorescence detection after 36 h. Supplementary Document S1: Sanger sequencing of QcMNCL-XIII0.1 gene. Supplementary Document S2: Sanger sequencing of *CACNA1G* gene.
