# Supplementary material for: Preparation and Functional Identification of a Novel Conotoxin QcMNCL-XIII0.1 from Conus quercinus
Source: Toxins (Basel). 2022 Jan 26;14(2):99. doi: 10.3390/toxins14020099 (PMC8877388; doi:10.3390/toxins14020099)
Supplement: Supplementary file 1 [file toxins-14-00099-s001.zip › toxins-1529340-supplementary.pdf]

Supplementary Materials: Preparation and Functional Identification of a Novel Conotoxin QcMNCL-XIII0.1 from *Conus quercinus*

Han Zhang, Anwen Liang and Xinghua Pan

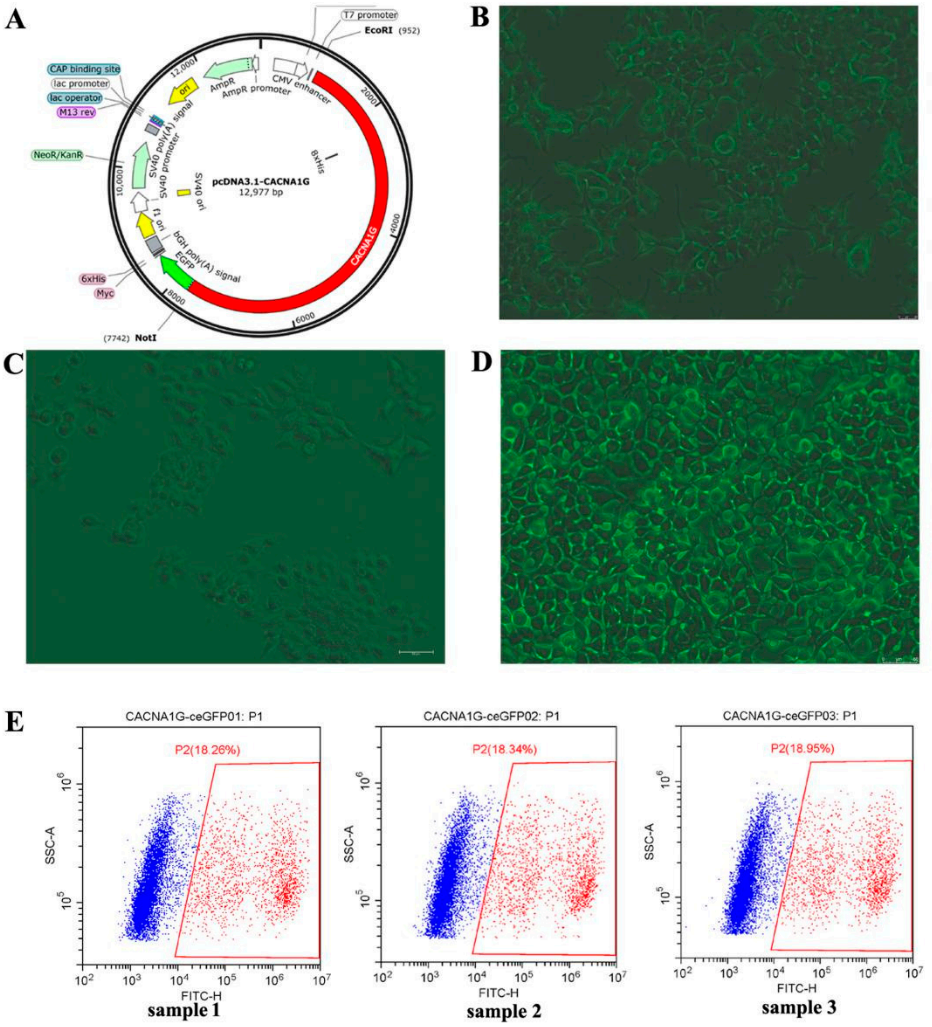

Figure S1. The 293T cells transfected with pcDNA3.1-CACNA1G-ceGFP detected by GFP fluorescence microscopy. (**A**), The map of pcDNA3.1-CACNA1G-ceGFP; (**B**), Fluorescence microscopic observation after 24 h; (**C**), Positive control group; (**D**), Fluorescence microscopic observation after 36 h; **E**, Flow cytometry fluorescence detection after 36 h.

Document S1 Sanger sequencing of QcMNCL-XIII0.1 gene.

(1) The map of the QcMNCL-XIII0.1 gene of constructed PET32a-QcMNCL-XIII0.1 expression vector and nucleic acid sequence.

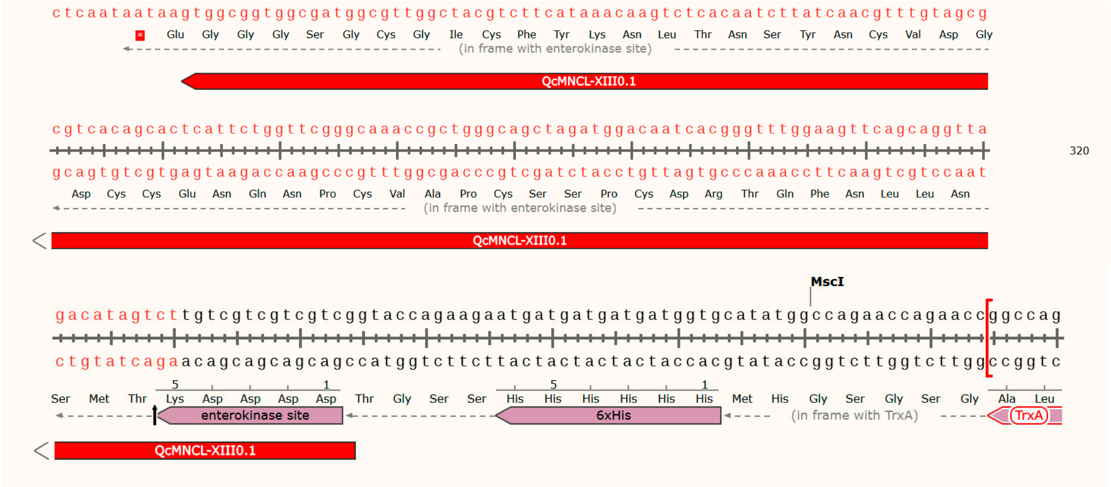

(2) Analysis and validation of original transcript sequence of the QcMNCL-XIII0.1 gene.

A. The nucleic acid sequence of the QcMNCL-XIII0.1 gene.

ACTATGTCTAACCTGCTGAACTTCCAAACCCGTGATTGTCCATCTAGCTGCCCAGCGGTTTG
CCCGAACCAGAATGAGTGCTGTGACGGCGATGTTTGCAACTATTCTAACACTCTGAACAAA
TACTTCTGCATCGGTTGCGGTAGCGGTGGCGGTGAATAATAACTCGAGCACCACCACCACC
ACCACTGAGATCCGGCTGCTAACAA

(3) Fluorescent peaks in the QcMNCL-XIII0.1 gene following Sanger sequencing.

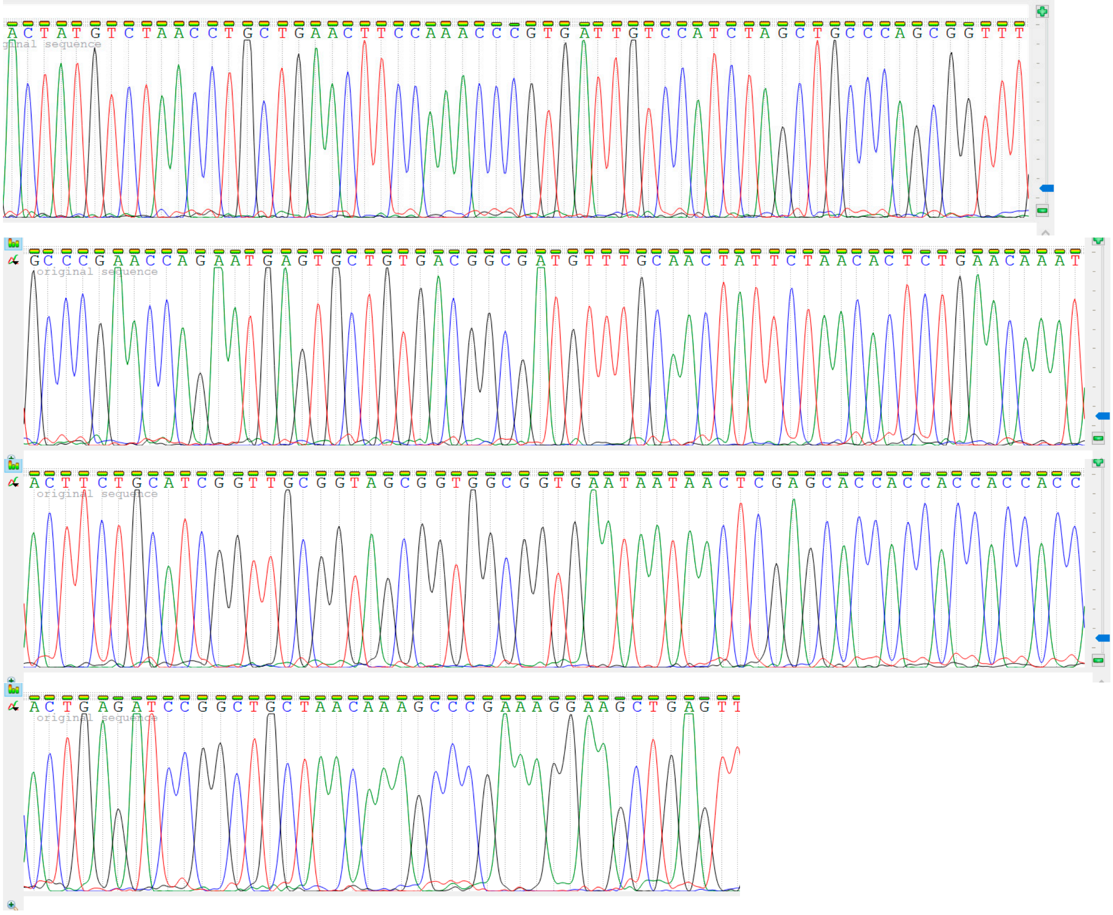

Document S2 Sanger sequencing of CACNA1G (6783bp) gene.

1. The 5-terminal sequencing of CACNA1G (6783bp) gene.

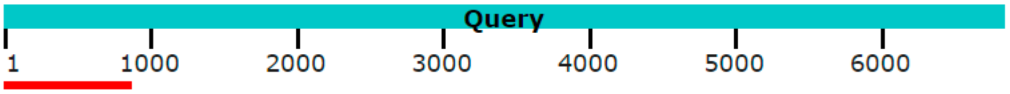

Sequence alignment between the 874bp nucleic acid sequence and CACNA1G (6783bp) gene.

The 874bp (red section) nucleic acid sequence of the CACNA1G gene.

ATGGACGAGGAGGAGGATGGAGCGGGCGCCGAGGAGTCGGGACAGCCCCGGAGCTTC
ATGCGGCTCAACGACCTGTCGGGGGCCGGGGGCCGGCCGGGGCCGGGGTCAGCAGAA
AAGGACCCGGGCAGCGCGGACTCCGAGGCGGAGGGGCTGCCGTACCCGGCGCTGGCC
CCGGTGGTTTTCTTCTACTTGAGCCAGGACAGCCGCCCGCGGAGCTGGTGTCTCCGCAC
GGTCTGTAACCCCTGGTTTGAGCGCATCAGCATGTTGGTCATCCTTCTCAACTGCGTGA
CCCTGGGCATGTTCCGGCCATGCGAGGACATCGCCTGTGACTCCCAGCGCTGCCGGATC
CTGCAGGCCTTTGATGACTTCATCTTTGCCTTCTTTGCCGTGGAGATGGTGGTGAAGAT
GGTGGCCTTGGGCATCTTTGGGAAAAAGTGTTACCTGGGAGACACTTGGAACCGGCTT
GACTTTTTCATCGTCATCGCAGGGATGCTGGAGTACTCGCTGGACCTGCAGAACGTCAG
CTTCTCAGCTGTCAGGACAGTCCGTGTGCTGCGACCGCTCAGGGCCATTAACCGGGTGC
CCAGCATGCGCATCCTTGTCACGTTGCTGCTGGATACGCTGCCCATGCTGGGCAACGTC
CTGCTGCTCTGCTTCTTCGTCTTCTTCATCTTCGGCATCGTCGGCGTCCAGCTGTGGGCA
GGGCTGCTTCGGAACCGATGCTTCCTACCTGAGAATTTCAGCCTCCCCCTGAGCGTGGA
CCTGGAGCGCTATTACCAGACAGAGAACGAGGATGAGAGCCCCTTCATCTGCTCCCAG
CCACGCGAGAACGGCATGCGGTCCTGCAGAAGCGTGCCCACGCTGCGCGGGGACG

2. Fluorescent peaks in the 874bp nucleic acid sequence of the CACNA1G gene following Sanger sequencing.

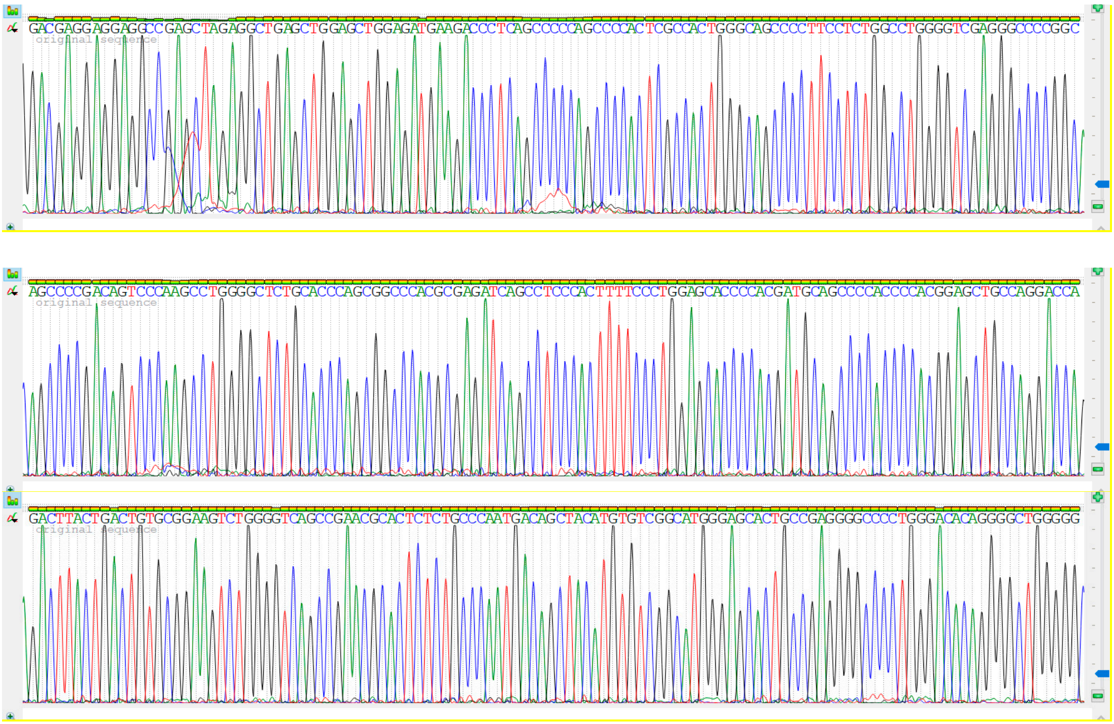

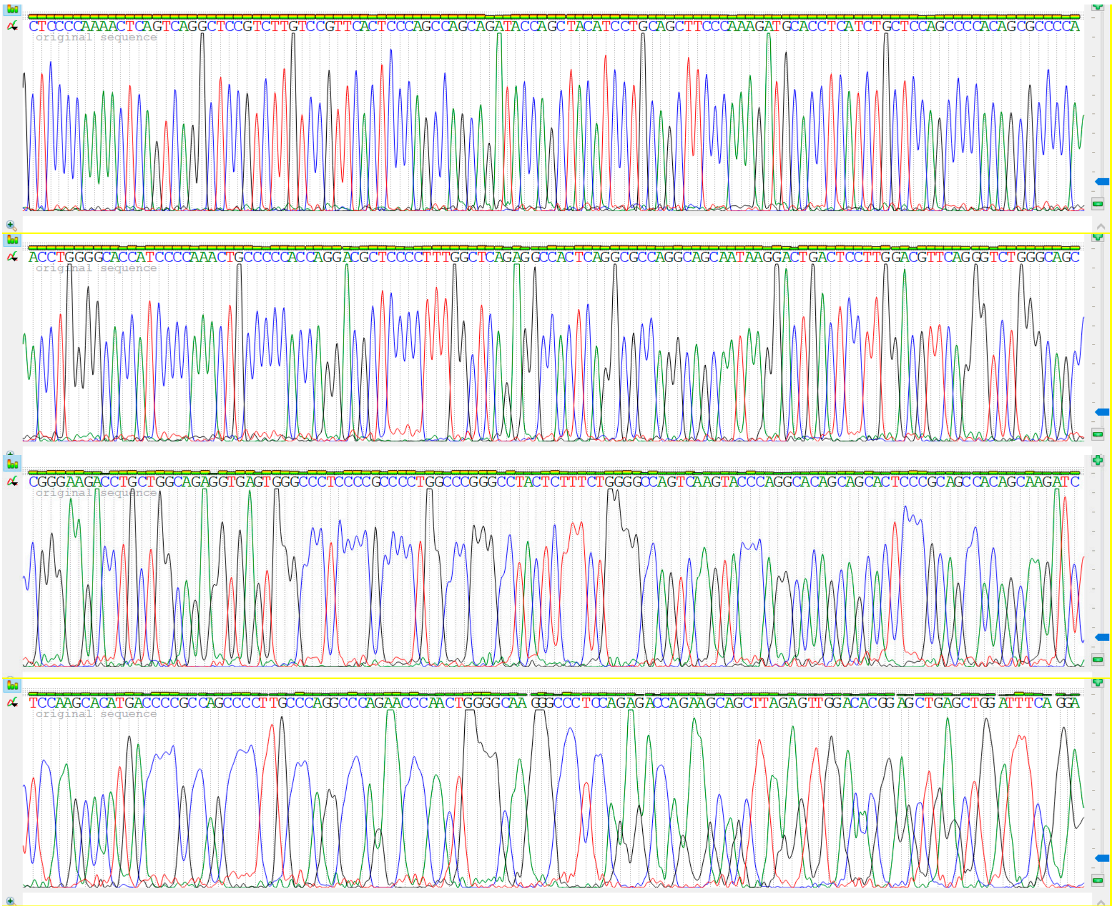

3. The 3-terminal sequencing of CACNA1G (6783bp) gene.

The 832bp (red section) nucleic acid sequence of the CACNA1G gene.

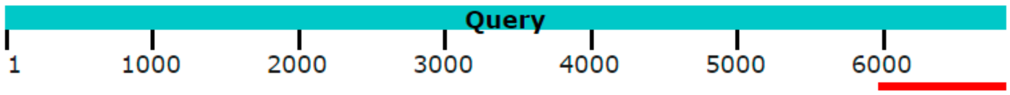

Sequence alignment between the 832bp nucleic acid sequence and CACNA1G gene.

TCATCTGCTCCAGCCCCACAGCGCCCCAACCTGGGGCACCATCCCCAAACTGCCCCCACCA
GGACGCTCCCCTTTGGCTCAGAGGCCACTCAGGCGCCAGGCAGCAATAAGGACTGACTCCT
TGGACGTTCAGGGTCTGGGCAGCCGGGAAGACCTGCTGGCAGAGGTGAGTGGGCCCTCCC
CGCCCCTGGCCCGGGCCTACTCTTTCTGGGGCCAGTCAAGTACCCAGGCACAGCAGCACTC
CCGCAGCCACAGCAAGATCTCCAAGCACATGACCCCGCCAGCCCCTTGCCCAGGCCCAGA
ACCCAACTGGGGCAAGGGCCCTCCAGAGACCAGAAGCAGCTTAGAGTTGGACACGGAGCT
GAGCTGGATTTCAGGAGACCTCCTGCCCCCTGGCGGCCAGGAGGAGCCCCCATCCCCACGG
GACCTGAAGAAGTGCTACAGCGTGGAGGCCCAGAGCTGCCAGCGCCGGCCTACGTCCTGG

CTGGATGAGCAGAGGAGACACTCTATCGCCGTCAGCTGCCTGGACAGCGGCTCCCAACCCC
ACCTGGGCACAGACCCCTCTAACCTTGGGGGCCAGCCTCTTGGGGGGCCTGGGAGCCGGCC
CAAGAAAAAACTCAGCCCGCCTAGTATCACCATAGACCCCCCCGAGAGCCAAGGTCCTCG
GACCCCGCCCAGCCCTGGTATCTGCCTCCGGAGGAGGGCTCCGTCCAGCGACTCCAAGGAT
CCCTTGGCCTCTGGCCCCCCTGACAGCATGGCTGCCTCGCCCTCCCCAAAGAAAGATGTGC
TGAGTCTCTCCGGTTTATCCTCTGACCCAGCAGACCTGGACCCC

Fluorescent peaks in the 832bp nucleic acid reverse complementary sequence of the CACNA1G gene following Sanger sequencing.

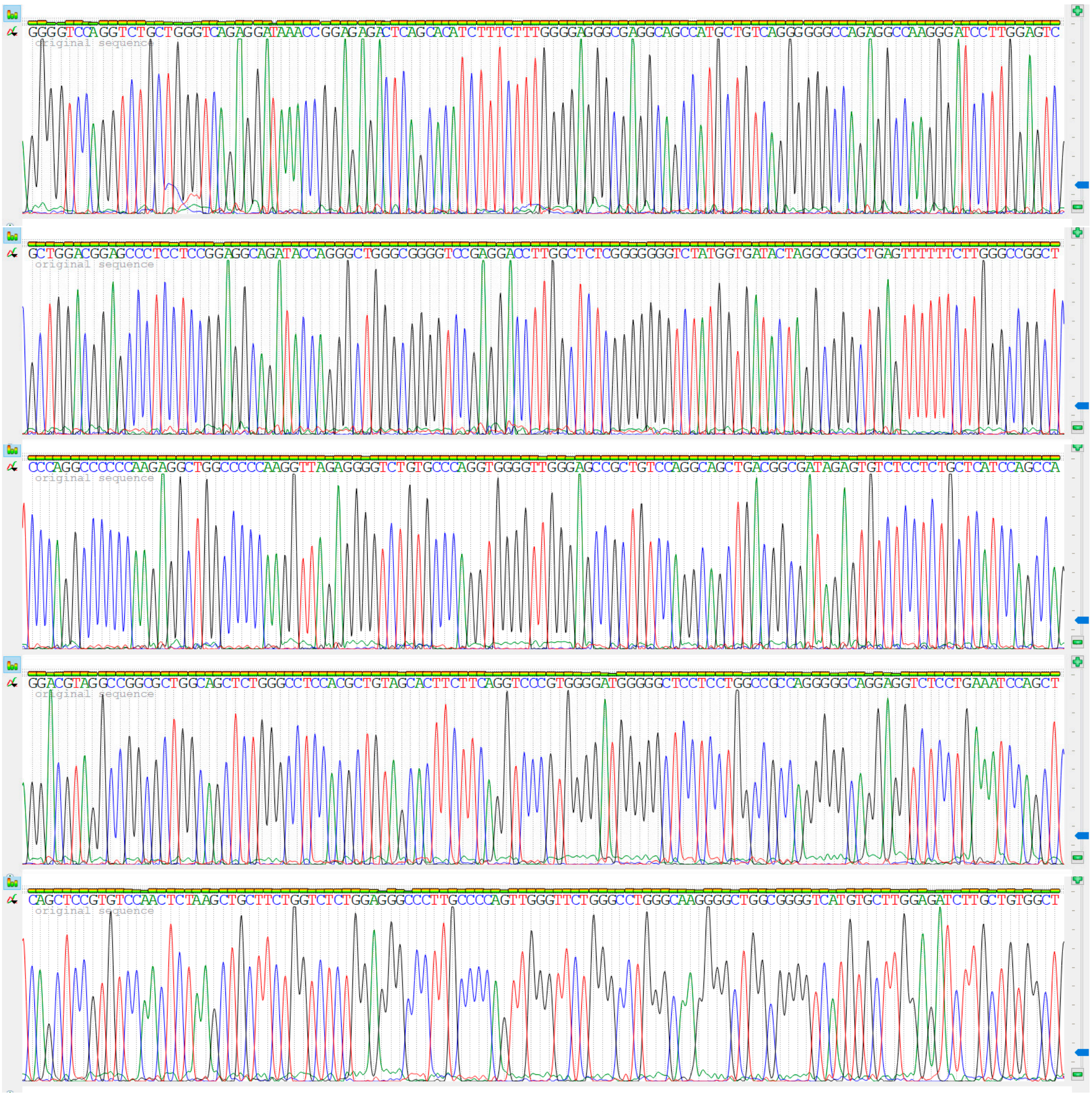

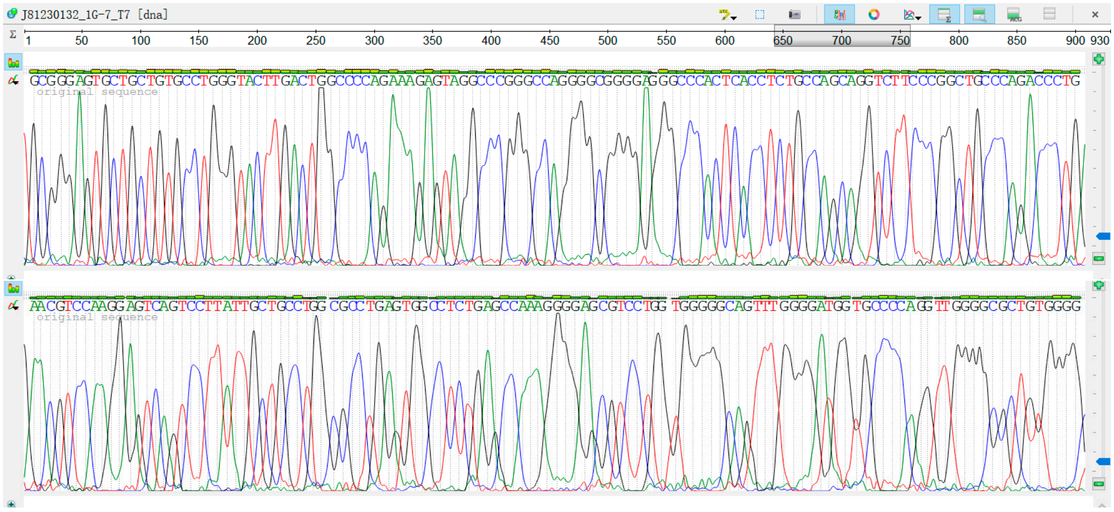

J81230132_1G-7_T7 [dna]
1 50 100 150 200 250 300 350 400 450 500 550 600 650 700 750 800 850 900 930
GCGGGAGTGCTGCTGTGCCTGGGTACTTGACTGGCCCCAGAAAGAGTAGGCCCGGGCCAGGGGCGGGGAGGGCCCACTCACCTCTGCCAGCAGGTCTTCCCGGCTGCCCAGACCCTG
original sequence
AACGTCCAAGGAGTCAGTCCTTATTGCTGCCTGG CGCCTGAGTGGCCTCTGAGCCAAAGGGGAGCGTCCTGG TGGGGGCAGTTTGGGGATGGTGCCCCAGGTTGGGGCGCTGTGGGG
original sequence
